# Social and Nonsocial Content Differentially Modulates Visual Attention and Autonomic Arousal in Rhesus Macaques

**DOI:** 10.1371/journal.pone.0026598

**Published:** 2011-10-26

**Authors:** Christopher J. Machado, Eliza Bliss-Moreau, Michael L. Platt, David G. Amaral

**Affiliations:** 1 Department of Psychiatry and Behavioral Sciences, California National Primate Research Center and The M.I.N.D Institute, University of California Davis, Davis, California, United States of America; 2 Department of Neurobiology, Center for Neuroeconomic Studies, Center for Cognitive Neuroscience, Duke University, Durham, North Carolina, United States of America; 3 Department of Evolutionary Anthropology, Duke University, Durham, North Carolina, United States of America; Kyushu University, Japan

## Abstract

The sophisticated analysis of gestures and vocalizations, including assessment of their emotional valence, helps group-living primates efficiently navigate their social environment. Deficits in social information processing and emotion regulation are important components of many human psychiatric illnesses, such as autism, schizophrenia and social anxiety disorder. Analyzing the neurobiology of social information processing and emotion regulation requires a multidisciplinary approach that benefits from comparative studies of humans and animal models. However, many questions remain regarding the relationship between visual attention and arousal while processing social stimuli. Using noninvasive infrared eye-tracking methods, we measured the visual social attention and physiological arousal (pupil diameter) of adult male rhesus monkeys (*Macaca mulatta*) as they watched social and nonsocial videos. We found that social videos, as compared to nonsocial videos, captured more visual attention, especially if the social signals depicted in the videos were directed towards the subject. Subject-directed social cues and nonsocial nature documentary footage, compared to videos showing conspecifics engaging in naturalistic social interactions, generated larger pupil diameters (indicating heightened sympathetic arousal). These findings indicate that rhesus monkeys will actively engage in watching videos of various kinds. Moreover, infrared eye tracking technology provides a mechanism for sensitively gauging the social interest of presented stimuli. Adult male rhesus monkeys' visual attention and physiological arousal do not always trend in the same direction, and are likely influenced by the content and novelty of a particular visual stimulus. This experiment creates a strong foundation for future experiments that will examine the neural network responsible for social information processing in nonhuman primates. Such studies may provide valuable information relevant to interpreting the neural deficits underlying human psychiatric illnesses such as autism, schizophrenia and social anxiety disorder.

## Introduction

Social cognition describes a collection of perceptual, cognitive and regulatory processes that coordinate one's interactions with others [1]–[5]. This brief definition does not capture the complexity of a process that humans, and even many nonhuman primate species [6], engage in effortlessly throughout each day. Research into how the primate brain normally processes social information, regulates emotional arousal and guides appropriate social behavior has flourished over the past three decades. Although much of this research has involved clinical and nonclinical human populations, studies using animal models, such as rhesus monkeys, have also provided many key insights including a list of brain regions that are putative components of the “social brain” [3]. Nonhuman primate studies using selective neurotoxic lesions ([7]–[12]), transient inactivation [13], functional neuroimaging [14]–[18] and electrophysiological recording techniques [19]–[32] have implicated the superior temporal cortex, amygdala, orbitofrontal cortex and anterior cingulate cortex in collectively decoding the meaning of social stimuli, orchestrating appropriate social and emotional responses and evaluating outcomes relative to predictions. These findings have largely corroborated data from human functional neuroimaging and lesion studies [33]–[35].

Despite the advances brought about through research on rhesus monkeys, this work comes with several caveats. First, most neurotoxic lesion and transient inactivation studies have measured social behavior deficits in operated and control animals while freely interacting in groups [7]–[9], [13]. While this affords the opportunity to record rich behavioral interactions, such studies allow for very little experimental control. Analyses of specific social information processing abilities in more controlled settings with such experimental groups could greatly aid the interpretation of observational data. Second, although electrophysiological recording and functional neuroimaging studies are conducted under highly-controlled conditions, these studies typically use static images of facial expressions or body postures as social stimuli. This is problematic because primate social communication using facial expressions and body postures involves motion and a sequential exchange between individuals, neither of which are adequately captured in a still image. High-quality video stimuli provide greater contextual and sequential information, and allow for behavioral responses to be measured away from extraneous physical, social or olfactory distractions [36], [37]. To date, video stimuli have only been used sparingly to study aspects of social information processing [38]–[42]. Macaques attend to videos depicting social stimuli for extended periods of time and viewing time is comparable for videos depicting aggression, affiliation and environmental exploration [38], [42]. Macaques also respond in a socially appropriate manner to videos. Male rhesus monkeys will, for example, produce appeasement gestures, avoid and gaze avert in response to videos of threatening males and will approach video clips of female conspecifics [38], [39], [42], [43]. Finally, while many scholars believe that emotional state and personality heavily influence how humans decode the meaning of social signals [44], few studies in nonhuman primates have measured peripheral physiological arousal in conjunction with social information processing.

In the current experiment, we measured visual social attention and sympathetic nervous system arousal in adult, male, rhesus monkeys as they watched a large library of social and nonsocial video clips. We measured visual attention using an infrared eye-tracking system and a noninvasive method of head restraint [45]. Dark-adapted pupil diameter was the index of sympathetic nervous system activity [46]–[48]. We hypothesized that animals would attend more to videos depicting species-typical social interactions or facial expressions than to nature documentary video footage showing non-primate animals or landscapes. We also predicted that videos with social content would result in larger pupil diameters indicating heightened automatic nervous system arousal relative to nonsocial nature documentary videos.

## Materials and Methods

### Ethics statement

All experimental procedures were noninvasive and developed in collaboration with the veterinary, animal husbandry and environmental enrichment staff at the California National Primate Research Center (CNPRC). All data presented here were collected at the CNPRC under a protocol (13483) approved by the University of California, Davis, Institutional Animal Care and Use Committee. All attempts were made (in terms of social housing, enriched diet, use of positive reinforcement strategies and minimizing the duration of daily training/testing sessions) to promote the psychological well-being of the animals in accordance with recommendations made by the Weatherall report, “The use of non-human primates in research.”

### Subjects and living conditions

Six adult male rhesus monkeys participated in this study. Each was born at the CNPRC and lived in 1 of 24, half-acre outdoor enclosures for at least 2 years before being relocated to indoor housing. Each of these outdoor enclosures contained approximately 70 animals of various ages and sexes. Once relocated indoors, each animal was housed in a standard adult macaque laboratory cage (66 cm width×61 cm length×81 cm height) with a minimum of 6 hours of socialization permitted with a neighboring animal each weekday. Depending on the relationship, daily socialization was either full, unrestrained interactions in either of the 2 adjoining cages or restricted to mostly visual interaction through a metal grate (moderate tactile access was also possible). The housing room was maintained on a 12-hour light/dark cycle. All animals were maintained on a diet of fresh fruit, vegetables and monkey chow (Lab Diet #5047, PMI Nutrition International Inc., Brentwood, MO), with water available *ad libitum*. The current study did not begin until the animals were 5.8–8.7 years old and weighed 10–14 kg.

### Training

Training methods and noninvasive head restraint strategies have been described in detail elsewhere [45]. Briefly, all training and subsequent data collection occurred while the animals sat in a modified primate chair with a slanted top (Crist Instrument Co., Inc., Damascus, MD). Head restraint was accomplished noninvasively using individualized thermoplastic helmets that could be affixed to the primate chair. Each animal was habituated to sitting in the primate chair with its helmet on for successively longer periods of time up to 90 minutes. Next, the animal's chair was rolled into a sound-attenuating testing chamber (Acoustic Systems, Austin, TX; 2.1 m wide×2.4 m tall×1.1 m deep) for habituation to this testing context and the video eye-tracker (Applied Science Laboratories, Bedford, MA; model R-HS-S6). A wide-screen, color video monitor (60.96 cm diagonal; Gateway Inc., Irvine, CA; model LP2424) was positioned at the monkey's eye level. The video monitor was positioned 127 cm from the animals' eyes, while the eye-tracking camera was positioned on a tripod 53.34 cm from the animals' eyes. A curved mouthpiece (Crist Instrument Co., Inc.; model # 5-RLD-00A) was attached to the top-left of the chair and connected to an automatic juice dispenser (Crist Instrument Co., Inc.; model # 5-RLD-E3) so that fluid reward could be dispensed throughout the testing session. A white noise generator (60 dB) inside of the chamber was used to mask outside auditory distractions.

Visual stimuli were presented to each monkey using a PC running the Eprime 2.0 Professional software package (Psychology Software Tools, Pittsburgh, PA). All gaze data were collected using the Eye-Trac 6 .NET User Interface program (Applied Science Laboratories) on a separate PC. Infrared luminance level, pupil threshold and corneal reflection threshold were set individually for each animal at the start of each session. Sampling rate for the infrared eye-tracking camera was set to 120 Hz. A standard nine-point calibration (3×3 matrix of calibration stimuli) was conducted prior to testing with each animal to ensure accuracy of gaze data collection. Calibration stimuli were videos presented in small portions of the screen (8.89×5.72 cm on screen, 4° visual angle) of rhesus monkeys from the outdoor housing enclosures at the CNPRC. The goal here was to attract the animal's gaze to different portions of the screen to calibrate the data acquisition software.

Once reliably calibrated, each monkey was trained to fixate color animated GIF images at random spatial positions on the computer screen for juice rewards that were manually dispensed by the experimenter. Animals completed this phase of training once they fixated the GIF images consistently for 2 consecutive days. During the second phase of fixation training, animals viewed either photographs (color or black and white; 5-second duration) on a 50% gray background or color video clips (taken from commercial movies and nature documentary DVDs; 30-second duration) on a black background. Each photograph or movie was separated by four 50% gray screens: 1) blank, 10-second duration, 2) black square target (3.4° visual angle) at center, 3) same black square target positioned randomly at 1 of 8 points around the screen periphery, and 4) blank, 10-second duration. Animals were required to fixate each black square target for at least 250 ms to obtain a small juice reward and move on to the next picture or movie trial, thus ensuring accuracy of the point-of-gaze data throughout a prolonged testing session. The animals completed the final phase of training once they finished 100 picture or 50 movie trials in less than 90 minutes on 3 consecutive days.

### Video catalog

A library of 600, 30-second color videos was created for this study. Half of these videos (300) were created from raw video footage of rhesus monkeys collected at the CNPRC. A majority of these “social” videos showed rhesus macaques from the CNPRC field cages engaging in social behaviors, such as aggression (25 videos), grooming (50 videos), play (50 videos), mounting (15 videos), foraging (50 videos) or sitting in groups without overt social behavior (nonspecific social behavior; 50 videos). These stimuli will be collectively referred to as Naturalistic Social videos. In addition, a series of videos was created that depicted camera- or subject-directed facial expressions of aggression or subordination (20 videos each), as well as videos of monkeys simply looking towards the camera but not producing any social signals (neutral subject directed; 20 videos). These stimuli will be collectively referred to as Subject Directed Social videos. The remaining 300 videos were gathered from commercial nature documentaries such as “Planet Earth” (BBC Warner, 2007) and “Life in the Undergrowth” (BBC Warner, 2006). These Nature videos depicted birds (53 videos), insects/invertebrates (43 videos), land mammals (62 videos), marine mammals/fish (57 videos) and landscapes/flowers (85 videos). None of the Nature, Naturalistic Social or Subject Directed Social videos showed humans, other nonhuman primate species, snakes or other natural predators of macaques (i.e., predatory cats or large reptiles).

Raw video footage was edited using the PowerDirector Express version 5.0 software (CyberLink Corp., Fremont, CA) on a PC. Each video clip only included footage depicting social behavior from a single category (e.g., grooming, aggressive subject directed, foraging, etc.) or animals from only one Nature category (e.g., land mammals, birds, insects, etc.). Given that the frequency of scene changes within a video may influence visual attention in rhesus monkeys [40], we created videos that included 1–7 equal segments or 0–6 scene changes (mode = 4 segments or 3 scene changes). Compiled clips were produced by the software in MPEG2 format. Two versions were made of each video in the catalog; 1 with a resolution of 720×480 (480p) and a second with 1280×720 (720p) resolution. The current study used the 720×480 resolution versions to maximize PC processing speed. All videos spanned 12.6° visual angle in the vertical direction and 23.2° visual angle in the horizontal plane. Examples of the Nature, Naturalistic Social and Subject Directed Social videos are provided online as supplementary material (see Videos S1, S2, S3, respectively).

### Video passive viewing task

Data described here were gathered over 12 days. On each test day, the animal was transported to the eye-tracking room, placed into the testing chair and its head was restrained with a thermoplastic helmet [45]. The animal's chair was then moved into the testing chamber, the mouthpiece for juice delivery was attached to the chair and the eye-tracker was calibrated as described above.

**Figure 1:**
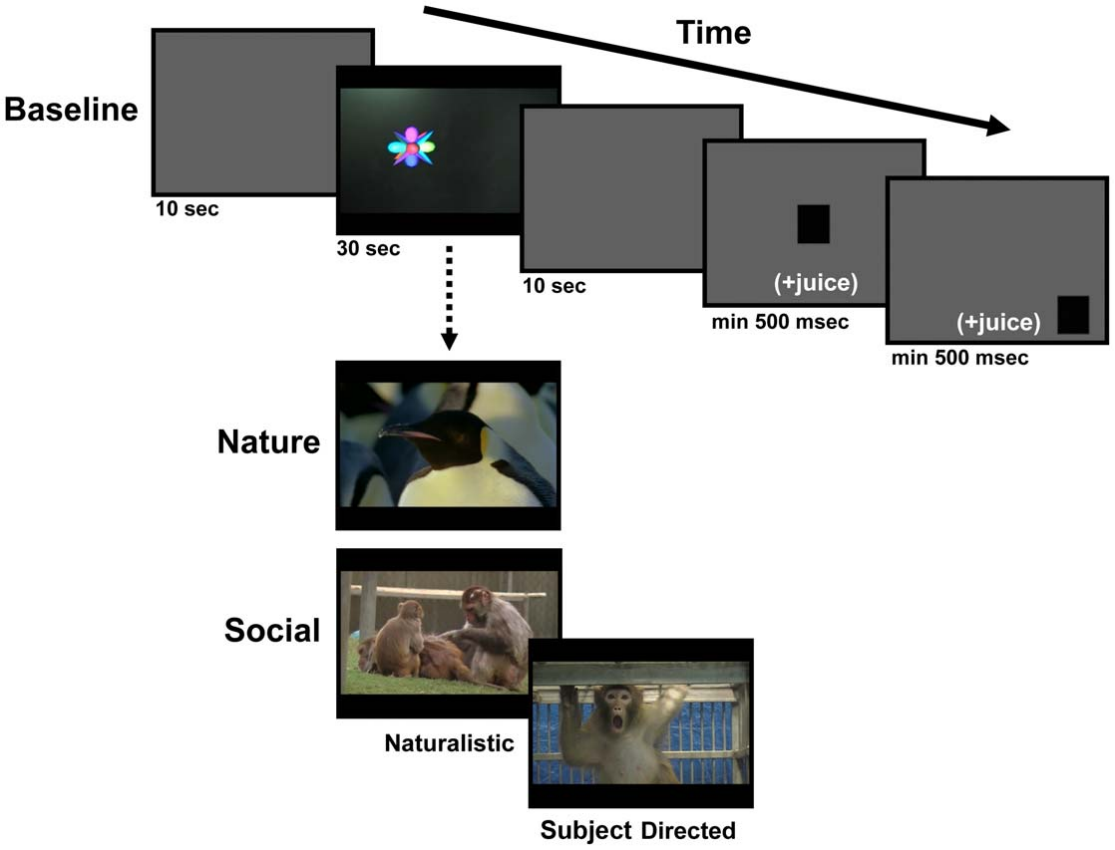
Schematic of a typical testing trial. A 10-second gray screen preceded and followed each 30-second video. During the first daily phase (Baseline; top panel), videos depicted simple, inanimate objects. During the second daily phase (bottom panel), animals viewed a pseudorandom mixture of Nature and Social (Naturalistic and Subject Directed) videos. After each video, animals were required to fixate a center and peripheral target for >500 ms to receive a juice reward and proceed to the next trial.

The first phase of a daily testing session required the animal to watch 10, 30-second “Baseline” videos that were made up of typical PC or Macintosh screen savers (e.g., Starfield, 3D FlowerBox, or 3D Pipes in Windows XP). These same 10 Baseline videos were shown in widescreen format (16∶9) and in random sequence each test day. Requiring the animals to watch these basic video stimuli served to verify eye-tracker calibration and the animal's willingness to participate in the testing session before the animal viewed the Naturalistic Social, Subject Directed Social and Nature videos of main interest. As shown at the top of Figure 1, each trial during the Baseline video session included the following sequence: 1) Blank gray screen that matched the average luminance of the subsequent video (10-second duration), 2) Baseline video (30-second duration), 3) Blank gray screen that matched the average luminance of preceding video (10-second duration), 4) Black square target (3.4° visual angle) at center of a 50% gray screen, 5) Same black square target positioned randomly at one of eight points around the 50% gray screen periphery. The animal was required to fixate the 2 black square targets for at least 500 ms to advance to the next trial and receive juice rewards (180 ms juice pulse for center target, 360 ms juice pulse for peripheral target).

In the second testing phase, the animal viewed a pseudorandom mixture of 25 Social and 25 Nature videos (Figure 1, bottom), also shown in widescreen format (16∶9). The Social videos included both Naturalistic Social and Subject Directed Social videos. The following 4 constraints were put on the daily sequence of videos: 1) Half of the test days began with a Social video and the remaining half began with a Nature video, 2) Five Social and 5 Nature videos occurred in each block of 10 videos, 3) No more than 2 Social or Nature videos could occur on consecutive trials, and 4) If 2 consecutive Social or Nature videos did occur, the 2 videos were from different categories (i.e., 2 grooming or land mammal videos were never presented consecutively). Each Social and Nature video was viewed only once by each animal during this study and all animals saw the videos in the same sequence each day. The Social and Nature videos were presented within the same trial structure as shown in Figure 1, with the same square target fixation requirements.

### Data analysis

Each animal's total fixation duration, total frequency of fixations, average fixation duration, average gaze dwell duration and average pupil diameter was measured for each video using the ASL Results software package (Applied Science Laboratories). Eye tracking parameters were computed for a single rectangular area of interest (AOI) that encompassed the entire video frame. Default parameters were used to define fixations (Applied Science Laboratories). A fixation was recorded if gaze coordinates remained within 1°×1° visual angle for at least 100 ms. The duration of a given fixation ended when gaze coordinates deviated by more than 1°×1° visual angle for more than 360 ms. Total fixation frequency represented the cumulative number of discrete fixations that fell within the video AOI during each 30-second trial. Total fixation duration was the cumulative time (maximum = 30 seconds) that the animal spent fixating the video AOI. The average fixation duration was derived by dividing the total fixation duration by the total fixation frequency. Finally, the average dwell duration measures the average amount of time that gaze remained within the video AOI without leaving.

We opted to normalize data within each animal to control for individual differences across test days. For each video, the animal's total fixation duration, total fixation frequency, average fixation duration or average dwell duration was divided by the average of that same variable across all 50 Social and Nature videos viewed that day. The resulting quotients were multiplied by 100 to give a metric that can be interpreted as a percent of the animal's typical total fixation duration, total fixation frequency, average fixation duration or average dwell duration on a given test day. As an example, if an animal fixated on a given video for 23 seconds, but their average fixation duration across all Social and Nature videos on that day was 18 seconds, then their normalized total fixation duration for the video in question was (23/18)×100 = 128 or 28% longer than the daily average fixation duration.

For each of these 4 dependent variables, we examined values for the entire 30-second video, as well as values for each 10-second block. Data were compared across broad categories of video stimuli (Nature, Subject Directed Social and Naturalistic Social), as well as within each category (i.e., Grooming vs. Play vs. Aggression, etc. in the Naturalistic Social category) using ANOVAs with Category (3–6) and Block (3) as within-subjects factors with repeated measures. A Huynh-Feldt correction was used to adjust the degrees of freedom if the group variance did not remain equal across the three 10-second blocks. Post-hoc *t*-tests of significant main effects and interactions were Bonferroni corrected for multiple comparisons. Alpha was set at *p*<.05, but significance levels up to *p* = .07 are also presented as marginally significant due to the small sample size and conservative approach to correction for multiple comparisons.

As an index of cognitive processing load [49], [50] and physiological arousal [51], [52] (especially reflecting sympathetic nervous system activation [46]–[48]) for each video, we also measured the animals' dark-adapted pupil diameter at 120 Hz. To remove data reflecting the pupillary light reflex, the first 2 seconds of each video trial were omitted from the analysis. Initial inspection of the video catalog revealed that the average luminance of the Nature videos was significantly lower than the average luminance of the Social videos (Naturalistic Social and Subject Directed Social combined; F(1,598) = 4.144; *p*<.05). Such a difference necessitated a normalization procedure to remove the influence of differences in luminance across video categories, as well as individual differences. We identified 2 normalization strategies. The gray screen that proceeded and followed each video had the same average luminance as that video. The raw average pupil diameter data from each video was therefore divided by the average pupil diameter during the gray screens flanking each video and multiplied by 100 to provide pupil diameters in terms of a percent of the gray screen value. An alternative approach was to use the same within-subject strategy as described for the gaze data above. The raw average pupil diameter for each video was divided by that animal's average pupil diameter across the entire testing session on that day and multiplied by 100 to provide pupil diameters in terms of a percent of the daily average. Analysis of the pupil diameter data was conducted using both normalization strategies and nearly identical results were found for both strategies. Therefore, for the sake of consistency with the gaze data, we will display results for the pupil diameter dataset using the within-subject normalization strategy. The results generated using the gray screen normalization strategy are included as Tables S1, S2, S3 online.

## Results

Examples of 1 Nature, Naturalistic Social and Subject Directed Social video are shown online with the pattern of visual attention (or point-of-gaze) from 1 animal mapped onto the video with black cross-hairs (Videos S4, S5, S6, respectively). The noninvasive thermoplastic helmets used for head restraint produced reliable gaze and pupil diameter data for all animals. Only 1.3% of the total trials (47 out of 3600) were discarded due to excessive head movement or other technical problems with the eye-tracker. The animals readily attended to the 600 videos. The 6 adult male rhesus monkeys spent an average of 21.19 seconds±1.81 SEM fixating on these videos, regardless of content. This average fixation duration was significantly higher than that of the Baseline videos (showing moving shapes) that were used during the session to verify eye-tracker calibration accuracy [*F*(1,5) = 14.74, *p* = .01, *η_p_^2^* = .747].

**Figure 2:**
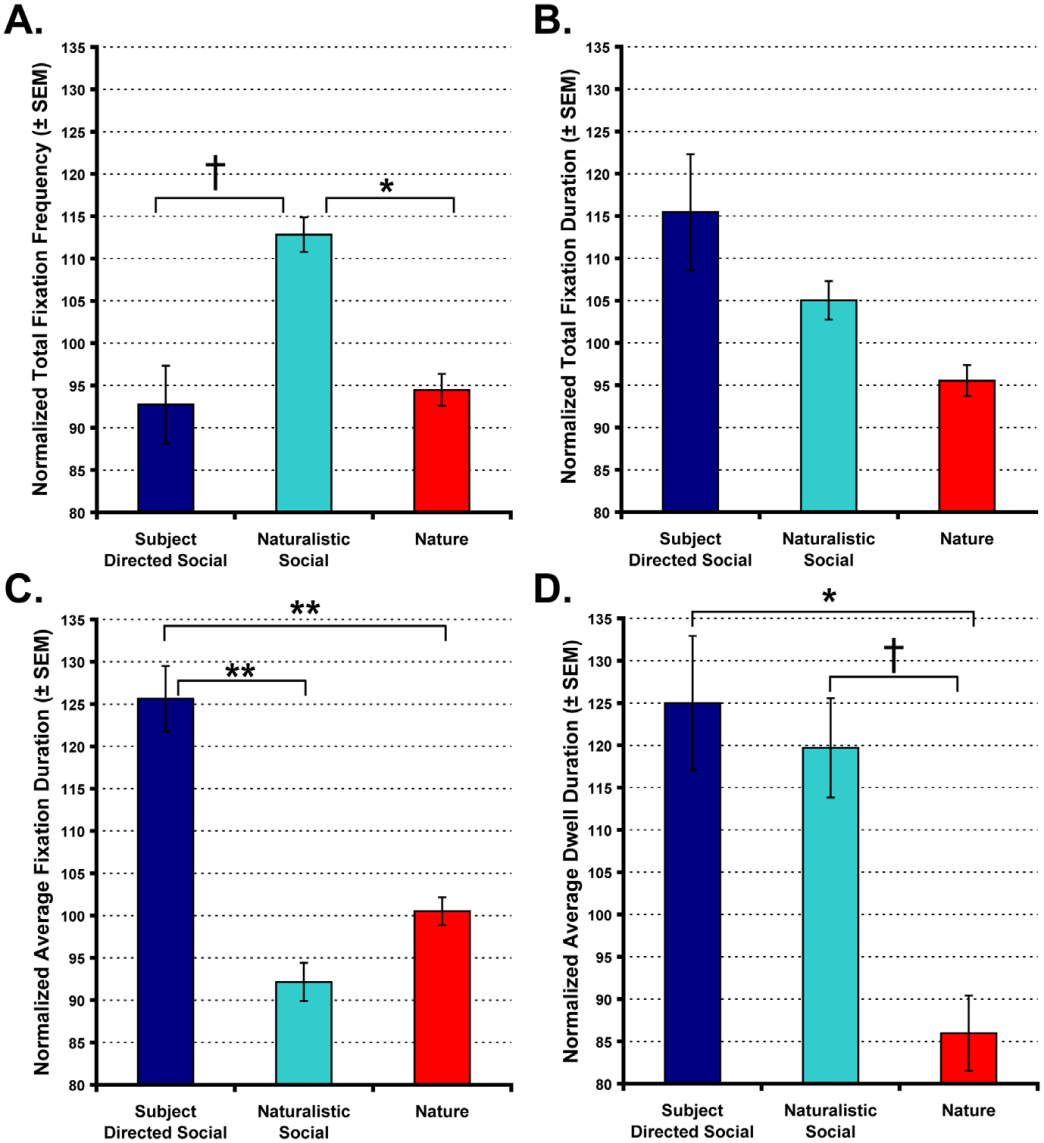
Rhesus monkeys discriminate social and nonsocial videos. Graphs show the total number of fixations (A), total fixation duration (B), average fixation duration (C) and average dwell duration (D) for the Subject Directed Social, Naturalistic Social and Nature video categories. Main effects of video category: † *p* = .07, * *p*<.05, ** *p*<.01. Vertical bars represent the standard error of the mean (SEM).

Based on the frequency of fixations, the monkeys' discriminated between the Nature, Naturalistic Social and Subject Directed Social video categories. As shown in Figure 2A, the animals fixated more frequently during for the Naturalistic Social videos than the Subject Directed Social or Nature videos [Category main effect; *F*(2,10) = 10.45, *p*<.01, *η_p_^2^* = .676; post-hoc: Naturalistic Social>Subject Directed Social *p* = .066, Naturalistic Social>Nature *p* = .015]. We also found that sub-categories within the Naturalistic Social videos differed in the total frequency of fixations [Category main effect; F(5,25) = 6.15, *p* = .001, *η_p_^2^* = .552]. In particular, the animals fixated more frequently when watching videos showing conspecifics engaged in mounting behavior relative to those showing foraging [post-hoc: Mounting>Foraging *p* = .021]. No other sub-categories within the Naturalistic Social videos differed significantly.

In contrast to the total number of fixations, Subject Directed Social videos commanded the greatest total fixation duration (Figure 2B), but the difference relative to the other 2 categories was not significant [Category main effect; *F*(2,10) = 4.685, *p* = .037, *η_p_^2^* = .484; post-hoc: Subject Directed Social = Naturalistic Social = Nature, all *p*>.11]. However, discrete fixations were significantly longer when animals watched Subject Directed Social videos [Figure 2C; Category main effect; *F*(2,10) = 31.406, *p*<.001, *η_p_^2^* = .863; post-hoc: Subject Directed Social>Naturalistic Social *p* = .002, Subject Directed Social>Nature *p* = .01]. More focused analysis of the 3 sub-categories within Subject Directed Social videos did not indicate any significant differences in average fixation duration based on type of content (i.e., aggressive, affiliative or neutral).

In addition to measures of gaze frequency and duration, we also measured the average time that the animals' gaze remained or “dwelled” within the video frame area of interest without leaving (Figure 2D). This measure showed that the Subject Directed Social and Naturalistic Social videos both captured the animals' attention for longer periods of time than the Nature videos [Category main effect; *F*(2,10) = 9.108, *p* = .006, *η_p_^2^* = .646; post-hoc: Subject Directed Social>Nature *p* = .036, Naturalistic Social>Nature *p* = .065]. There was no significant difference in average dwell duration between the sub-categories of Subject Directed Social videos. However, within the Naturalistic Social category, aggression and mounting videos captured the animals' attention for longer periods of time than foraging videos [Category main effect; *F*(5,25) = 15.674, *p*<.001, *η_p_^2^* = .758; post-hoc: Aggression>Foraging *p* = .016, Mounting>Foraging *p* = .005]. Finally, the measures of total fixation frequency, total fixation duration, average fixation duration and average dwell duration were all consistent throughout the entire video (i.e., there were not significant main effects of Block), indicating that these measures of visual attention were not consistently driven by 1 particular segment of the videos.

**Figure 3:**
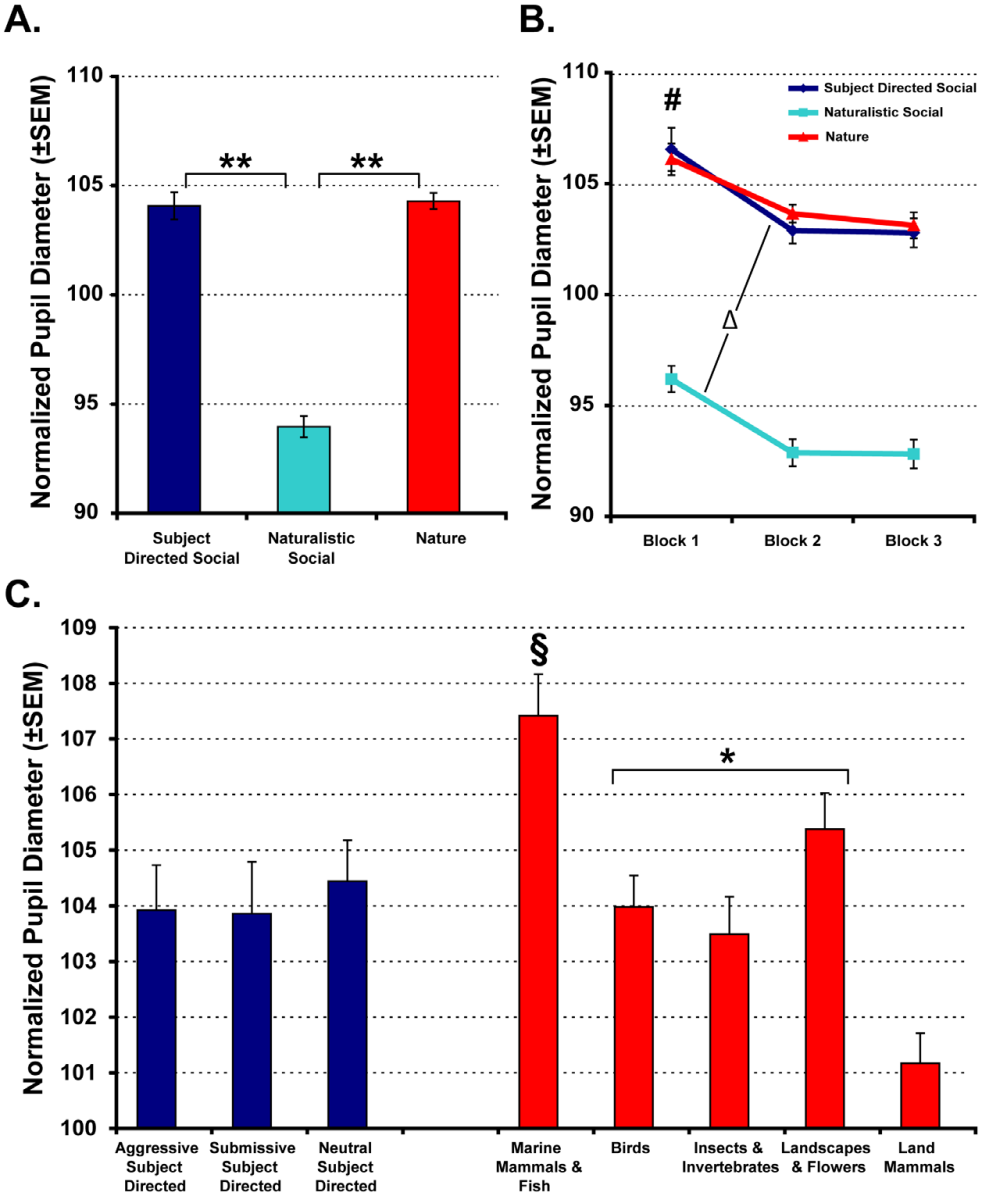
Sympathetic arousal, as indexed by pupil diameter, varies with video content. Within-subject normalized pupil diameter data shown for the entire 30-second video (A) or when videos were analyzed in 10-second blocks (B). Pupil diameters are also shown for specific sub-categories within the Subject Directed Social and Nature categories (C). Main effects of video category: § *p*<.05 relative to all other Nature subcategories, * *p*<.05 relative to Land Mammals only, ** *p*<.01. Main effects of time block: # *p*<.05. Change between Block 1 and Block 2: Δ *p*<.05. Vertical bars represent the standard error of the mean (SEM).

Average pupil diameter during each video was also collected to provide a measure of sympathetic nervous system activity [46]–[48]. As shown in Figure 3A, mean pupil diameter was significantly larger when the animals watched the Subject Directed Social and Nature videos relative to the Naturalistic Social videos [Category main effect; *F*(2,10) = 101.53, *p*<.001, *η_p_^2^* = .953; post-hoc: Subject Directed Social and Nature>Naturalistic Social, both *p*<.001]. Pupil diameter was also largest during the first 10 seconds of a given video (Block 1 of 3), regardless of content [Block main effect; *F*(2,10) = 14.364, *p* = .01, *η_p_^2^* = .742; post-hoc: Block 1>Block 2 *p*<.05, Block 1>Block 3 *p* = .05; Figure 3B]. Finally, there was a significant Category×Block interaction. For Subject Directed Social and Naturalistic Social videos, pupil diameter decreased significantly between Blocks 1 and 2 [*t*(5) = 4.8 and 5.1, respectively, both *p*<.05], but remained static between Blocks 2 and 3. By contrast, when animals watched Nature videos, their pupil diameters decreased slightly across the 30-second video, but did not change significantly. We also examined the sub-categories within the Subject Directed Social and Nature videos to determine if 1 or more specific type of content could be driving the larger pupil diameters observed for the more general category. There were no significant differences in pupil diameter between the 3 sub-categories of Subject Directed Social videos (Figure 3C). However, within the Nature video category, pupil diameter was significantly greater when the animals watched videos showing marine mammals and fish relative to all other sub-categories (all *p*<.05). Pupil diameter was intermediate and did not differ between videos showing birds, insects/invertebrates and landscapes/flowers, but each of these 3 sub-categories still produced pupil diameters greater than land mammals (all *p*<.05 relative to land mammal sub-category).

## Discussion

The present study assessed how visual attention and autonomic nervous system arousal are differentially modulated by social and nonsocial information. This was assessed using noninvasive video eye-tracking and pupillometry to simultaneously measure differences in visual attention and sympathetic nervous system arousal when adult male rhesus monkeys watched high-quality videos. Overall, adult male rhesus monkeys fixated more frequently and for longer durations when watching videos with social content relative to nonsocial, nature documentary footage. Relative to nature documentary footage, videos showing macaque social signals directed towards the viewer commanded the longest average fixation duration, total fixation duration and average duration of continuous fixation or “dwell” within the video frame. Videos showing conspecifics engaged in naturalistic social interactions resulted in the highest total number of fixations, but videos in this category typically fell between those showing subject-directed social signals or nature documentary footage in fixation duration measures of attention (i.e., average dwell duration).

These results confirmed our expectations that were based on previous eye-tracking studies with humans, apes and monkeys indicating a strong preference for attending to social stimuli, especially faces [41], [42], [53]–[62]. In particular, the 2 kinds of social videos (Naturalistic and Subject Directed) produced similar values for total fixation duration and average dwell duration, and these values were greater than those measured for the Nature videos. It is therefore unlikely that differences in raw motion (i.e., activity of the stimuli in the video) could account for this pattern of results, since the amount of overall motion was typically high for the Naturalistic Social and Nature videos, and significantly less for the Subject Directed Social videos. For these 2 measures, the pattern of results seems to be more driven by species-typical social content than overall motion. By contrast, average fixation duration was greater for the Subject Directed Social videos, relative to the Naturalistic Social and Nature videos. This pattern of results does suggest that overall motion may have contributed to average fixation duration, with higher levels of motion resulting in fixations that, on average, last for shorter periods of time. This pattern of results has also been observed in humans [63], [64]. The elevated number of fixations for naturalistic social videos relative to the subject-directed social or nature documentary footage was an unexpected finding. This may have been due to the fact that the naturalistic social behavior videos had more background stimuli overall (other monkeys moving around in the large housing enclosures at the CNPRC) than either the Subject Directed Social videos (plain blue or white background) or the Nature videos (naturalistic backgrounds, but a heavy focus on animals featured in the clip). As such, there were simply more discrete stimuli of interest on which to fixate during the naturalistic social videos than in the other video categories.

As is common for most infrared eye-tracking systems currently available, we were also able to measure 1 index of autonomic nervous system arousal in our animals – pupil diameter. The diameter of the human and nonhuman primate pupil is controlled by muscles in the iris that contract and relax [65], [66]. Parasympathetic nervous system fibers innervate the sphincter pupillae of the iris, and their activity results in pupillary constriction. By contrast, increased activity of sympathetic nervous system innervation to the dilator pupillae of the iris results in pupillary dilation [65], [66]. When a visual stimulus is presented, a rapid (1–2 second) constriction of the pupil occurs. This phenomenon is called the pupillary light reflex and its magnitude and latency varies depending on the overall brightness of the stimulus [67]–[70]. To decrease the effect of stimulus brightness on our results, we omitted the first 2 seconds of pupil diameter data from each video trial. Throughout the presentation of a particular visual stimulus, pupil diameter fluctuates depending on many factors, including cognitive processing load [49], [50] and physiological arousal [51], [52]. Pupil diameter also typically diminishes over time during the presentation of a stimulus, as the subject habituates, or with fatigue across a testing session [65], [66].

In contrast to the clear visual preference for videos with conspecific social content, we showed here that both subject-directed social signals and nature documentary footage, relative to those with naturalistic social interaction content, resulted in larger pupil diameters indicating elevated sympathetic nervous system arousal. The elevated autonomic response in the subject-directed videos was expected. Although the subject-directed social videos varied in the type of social signals displayed (agonistic, submissive or neutral expressions), all consisted of close-up footage of unfamiliar monkeys displaying direct eye contact toward the camera/viewer. In rhesus monkey society, direct eye-contact is a highly threatening gesture and readily elicits behavioral expressions of fear, passive avoidance and/or generalized tension [71], [72]. Acute fear or generalized anxiety have been linked for some time to physiological changes in general, and to sympathetic nervous system activation in particular (see for review [73]). Therefore, it is likely that the direct eye contact seen in this video category contributed to the increased pupil diameters measured for these videos relative to the naturalistic social videos which showed less provocative general social behavior.

The increased pupillary response to the Nature category of videos was not expected. One possible explanation for the elevated pupil diameters could be the novelty of the content in those videos. The monkeys used in this study were raised in semi-naturalistic outdoor enclosures. As a result, they had limited exposure to non-primate animals (such as birds, other small mammals and insects) during development. The rural landscape in which the housing enclosures are embedded is very consistent. Thus, the marine mammals or fish, as well as the oceanic, forest, desert and polar landscapes shown in the nature documentary footage were highly novel to our subjects. There was evidence of habituation in the temporal characteristics of pupil diameters (significantly lower pupil diameters after the first 10 seconds) for the Subject Directed and Naturalistic social videos, but not for the Nature videos (no change in pupil diameter across the 30-second video; Figure 3B) which further suggests they were highly novel. Consistent with the idea that increased arousal observed during the Nature clips resulted from novelty is the finding that videos that included marine mammals and fish resulted in the highest average pupil diameter (Figure 3B). The landscapes and flowers subcategory produced the second-largest average pupil diameters. In fact, videos showing birds, insects or landscapes all produced pupil diameters that were significantly larger than videos showing land mammals (i.e., animals most similar in body structure and locomotion to our subjects). Similar to acute fear or anxiety discussed above, novelty has also been shown to result in elevated peripheral sympathetic arousal in the rat [74], [75], monkey [76] and human [77], [78]. Therefore, it is plausible to conclude that the sustained elevations in pupil diameter observed for the nature documentary videos was driven by the overall novelty of the animals and locations displayed in these stimuli.

An alternative explanation for this pattern of results could be that the Nature videos, and particularly the ones showing marine mammals and fish, were darker than other video categories and it was that factor that drove the heightened pupil diameters. This is a plausible explanation, but unlikely given how the pupil diameter data were normalized. One strategy computed each animal's pupil diameters as a percentage of the average pupil diameter measured across the 25 social and 25 nonsocial videos shown on that particular day (within-subject normalization strategy; see Methods and Results sections). These data were also analyzed after expressing each animal's pupil diameter as a percentage of the diameter recorded during a gray screen that preceded and followed each video and had the same average luminance as that video (gray screen normalization strategy; see Tables S1, S2, S3 online). A very consistent pattern of results emerged regardless of which method was used to correct pupil diameters for differences in luminance across videos. Nature and Subject Directed Social videos result in greater pupil diameters relative to Naturalistic Social videos, and videos showing marine mammals and fish tend to produce the largest pupil diameters within the Nature category. This finding does not entirely exclude the possibility that brightness is playing a role in modulating pupil diameter, but it is unlikely to be the major driving factor.

Beyond the novel results produced by this study, we also have introduced a valuable library of videos that can now be used to study various aspects of social cognition in rhesus monkeys. A total of 600, 30-second color videos were made, half of which showed rhesus macaques engaged in various forms of social behavior or displaying social gestures indicative of aggression or subordination. The remaining half of the stimulus pool showed landscapes and fauna, or other, non-primate animals. The number of scenes or camera position transitions varied consistently according to a normal distribution within each category of Social or Nature video. For all videos created from raw footage collected at the CNPRC, measures were taken to eliminate any opaque barriers (fencing or cage bars) between the animals and the video camera. Such an extensive stimulus set does not currently exist in the fields of social neuroscience or primatology, and therefore may be of value to other investigators. For example, the normative point-of-gaze and pupil diameter data already generated in the current study could be used to select specific stimuli for future studies. These stimuli could also be used during functional neuroimaging or electrophysiological recording studies, alone or in conjunction with static images, to ask questions about how the primate brain represents social context or sequential information during social interactions. These video stimuli will be made available to other investigators and should be requested from the corresponding author.

In summary, the study of social neuroscience, like other areas of neuroscience, can benefit greatly from comparative studies in humans and animal models. This is especially true if studies utilize stimuli that closely reflect naturalistic conditions and use similar methods (such as eye-tracking and pupillometry) across species. Understanding how different kinds of experimental manipulations (e.g., brain lesions, transient deactivation or hyper-activation of a given brain structure, atypical prenatal or post-natal environment, etc.) perturb specific aspects of social cognition (perception, evaluation, motivation and behavioral/physiological regulation) will greatly facilitate the development of new insights into the underlying neurobiology of human psychiatric illnesses.

## Supporting Information

Table S1 **Pupil diameter analysis using the gray screen normalization method and comparing the three main video categories.**
(DOCX)

Table S2 **Pupil diameter analysis using the gray screen normalization method and comparing the Subject Directed Social video sub-categories.**
(DOCX)

Table S3 **Pupil diameter analysis using the gray screen normalization method and comparing the Nature video sub-categories.**
(DOCX)

Video S1 **A sample video from the Nature video category.**
(MOV)

Video S2 **A sample video from the Naturalistic Social video category.**
(MOV)

Video S3 **A sample video from the Subject Directed Social video category.**
(MOV)

Video S4 **A sample video from the Nature video category with black cross-hairs superimposed to indicate one animal's point-of-gaze.**
(MOV)

Video S5 **A sample video from the Naturalistic Social video category with black cross-hairs superimposed to indicate one animal's point-of-gaze.**
(MOV)

Video S6 **A sample video from the Subject Directed Social video category with black cross-hairs superimposed to indicate one animal's point-of-gaze.**
(MOV)
