# Correction: Social and Nonsocial Content Differentially Modulates Visual Attention and Autonomic Arousal in Rhesus Macaques

**DOI:** 10.1371/annotation/7e497d79-eabd-4345-989f-ea530dfb6ac0

**Published:** 2013-01-08

**Authors:** Christopher J. Machado, Eliza Bliss-Moreau, Michael L. Platt, David G. Amaral

The original version of the supplementary file S1 could no longer be displayed online due to copyright-related restrictions. That video has been replaced with a similar video from the same category (i.e., birds) created from footage acquired by the first author. The videos in the supplementary files S4, S5 and S6 have also been replaced with higher-quality versions created with an updated version of the ASL Results Plus software that was not available when this article was first published. 

