# Supplementary material for: Social and Nonsocial Content Differentially Modulates Visual Attention and Autonomic Arousal in Rhesus Macaques
Source: PLoS One. 2011 Oct 26;6(10):e26598. doi: 10.1371/journal.pone.0026598 (PMC3202553; doi:10.1371/journal.pone.0026598)
Supplement: Table S1 — Pupil diameter analysis using the gray screen normalization method and comparing the three main video categories. (DOCX) [file pone.0026598.s001.docx]

**Supplementary Table S1 – Pupil diameter analysis using gray screen normalization: Comparison of main video categories**

| **Comparison** | **Mean** | **Standard Error** | ***t*** | **df** | **Significance**  **(2 tailed & Bonferroni Corrected)** |
| --- | --- | --- | --- | --- | --- |
| Subject Directed Social | 94.55 | 1.20 |  |  |  |
| vs. |  |  | 13.21 | 5 | p < .001 |
| Naturalistic Social | 86.85 | 1.52 |  |  |  |
| Subject Directed Social | 94.55 | 1.20 |  |  |  |
| vs. |  |  | 1.22 | 5 | p = .828 |
| Nature | 95.40 | 1.36 |  |  |  |
| Naturalistic Social | 86.85 | 1.52 |  |  |  |
| vs. |  |  | 10.28 | 5 | p < .001 |
| Nature | 95.40 | 1.36 |  |  |  |

Data are the means, standard errors and *t*-test results for comparisons between the three main video categories used in this study.
