# Supplementary material for: Social and Nonsocial Content Differentially Modulates Visual Attention and Autonomic Arousal in Rhesus Macaques
Source: PLoS One. 2011 Oct 26;6(10):e26598. doi: 10.1371/journal.pone.0026598 (PMC3202553; doi:10.1371/journal.pone.0026598)
Supplement: Table S2 — Pupil diameter analysis using the gray screen normalization method and comparing the Subject Directed Social video sub-categories. (DOCX) [file pone.0026598.s002.docx]

**Supplementary Table S2 – Pupil diameter analysis using gray screen normalization: Comparison of Subject Directed Social video sub-categories**

| **Comparison** | **Mean** | **Standard Error** | ***t*** | **df** | **Significance**  **(2 tailed & Bonferroni Corrected)** |
| --- | --- | --- | --- | --- | --- |
| Aggressive Subject Directed | 94.68 | .97 |  |  |  |
| vs. |  |  | .98 | 5 | p = 1.0 |
| Neutral Subject Directed | 95.25 | 1.53 |  |  |  |
| Aggressive Subject Directed | 94.68 | .97 |  |  |  |
| vs. |  |  | 2.49 | 5 | p = .165 |
| Submissive Camera Directed | 93.73 | 1.14 |  |  |  |
| Neutral Subject Directed | 95.25 | 1.53 |  |  |  |
| vs. |  |  | 2.96 | 5 | p = .093 |
| Submissive Camera Directed | 93.73 | 1.14 |  |  |  |

Data are the means, standard errors and *t*-test results for comparisons between the three sub-categories of the Subject Directed Social video category.
