# Supplementary material for: Social and Nonsocial Content Differentially Modulates Visual Attention and Autonomic Arousal in Rhesus Macaques
Source: PLoS One. 2011 Oct 26;6(10):e26598. doi: 10.1371/journal.pone.0026598 (PMC3202553; doi:10.1371/journal.pone.0026598)
Supplement: Table S3 — Pupil diameter analysis using the gray screen normalization method and comparing the Nature video sub-categories. (DOCX) [file pone.0026598.s003.docx]

**Supplementary Table S3 – Pupil diameter analysis using gray screen normalization: Comparison of Nature video sub-categories**

| **Comparison** | **Mean** | **Standard Error** | ***t*** | **df** | **Significance**  **(2 tailed & Bonferroni Corrected)** |
| --- | --- | --- | --- | --- | --- |
| Birds | 95.48 | 1.55 |  |  |  |
| vs. |  |  | 1.74 | 5 | p = 1.0 |
| Insects & Invertebrates | 94.35 | 1.22 |  |  |  |
| Birds | 95.48 | 1.55 |  |  |  |
| vs. |  |  | 1.82 | 5 | p = 1.0 |
| Landscapes & Flowers | 96.36 | 1.25 |  |  |  |
| Birds | 95.48 | 1.55 |  |  |  |
| vs. |  |  | 4.42 | 5 | p = .70 |
| Land Mammals | 94.05 | 1.60 |  |  |  |
| Birds | 95.48 | 1.55 |  |  |  |
| vs. |  |  | 4.59 | 5 | p = .60 |
| Marine Mammals & Fish | 96.77 | 1.32 |  |  |  |
| Insects & Invertebrates | 94.35 | 1.22 |  |  |  |
| vs. |  |  | 4.33 | 5 | p = .80 |
| Landscapes & Flowers | 96.36 | 1.25 |  |  |  |
| Insects & Invertebrates | 94.35 | 1.22 |  |  |  |
| vs. |  |  | .388 | 5 | p = 1.0 |
| Land Mammals | 94.05 | 1.60 |  |  |  |
| Insects & Invertebrates | 94.35 | 1.22 |  |  |  |
| vs. |  |  | 4.37 | 5 | p = .70 |
| Marine Mammals & Fish | 96.77 | 1.32 |  |  |  |
| Landscapes & Flowers | 96.36 | 1.25 |  |  |  |
| vs. |  |  | 4.18 | 5 | p = .90 |
| Land Mammals | 94.05 | 1.60 |  |  |  |
| Landscapes & Flowers | 96.36 | 1.25 |  |  |  |
| vs. |  |  | 1.35 | 5 | p = 1.0 |
| Marine Mammals & Fish | 96.77 | 1.32 |  |  |  |
| Land Mammals | 94.05 | 1.60 |  |  |  |
| vs. |  |  | 5.77 | 5 | p = .02 |
| Marine Mammals & Fish | 96.77 | 1.32 |  |  |  |

Data are the means, standard errors and *t*-test results for comparisons between the five sub-categories of Nature videos used in this study.
